# Autism and the right to education in the EU: policy mapping and scoping review of Nordic countries Denmark, Finland, and Sweden

**DOI:** 10.1186/s13229-019-0290-4

**Published:** 2019-12-11

**Authors:** Robin van Kessel, Sebastian Walsh, Amber N. V. Ruigrok, Rosemary Holt, Anneli Yliherva, Eija Kärnä, Irma Moilanen, Eva Hjörne, Shruti Taneja Johansson, Diana Schendel, Lennart Pedersen, Meta Jørgensen, Carol Brayne, Simon Baron-Cohen, Andres Roman-Urrestarazu

**Affiliations:** 1 Department of International Health, School CAPHRI, Faculty of Health, Medicine and Life Sciences, Maastricht University, Maastricht, the Netherlands; 2 Institute of Public Health, University of Cambridge, Cambridge, UK; 3 Autism Research Centre, Department of Psychiatry, University of Cambridge, Cambridge, UK; 4 Child Language Research Center, Logopedics, Faculty of Humanities, University of Oulu, Oulu, Finland; 5 Philosophical Faculty, School of Educational Sciences and Psychology, University of Eastern Finland, Joensuu, Finland; 6 PEDEGO, University of Oulu, Oulu, Finland; 7 Child Psychiatric Clinic, University Hospital of Oulu, Oulu, Finland; 8 Department of Education and Special Education, University of Gothenburg, Gothenburg, Sweden; 9 iPSYCH, The Lundbeck Foundation Initiative for Integrative Psychiatric Research, Aarhus, Denmark; 10 National Centre for Register-Based Research, Aarhus University, Aarhus, Denmark; 11 Department of Public Health, Aarhus University, Aarhus, Denmark; 12 Center for Autisme, Herlev, Denmark; 13 Special Area Autism, Central Region, Aarhus, Denmark

## Abstract

**Introduction:**

The universal right to education for people with disabilities has been highlighted by the Universal Declaration on Human Rights and the Convention on the Rights of Persons with Disabilities. In this paper, we mapped policies addressing the right to education and special education needs of autistic children in Denmark, Sweden, and Finland**.**

**Methods:**

A policy path analysis was carried out using a scoping review as an underlying framework for data gathering. Policy mapping was performed independently by both lead authors to increase reliability.

**Results and discussion:**

The values of the Universal Declaration of Human Rights and the Convention on the Rights of Persons with Disabilities have been closely translated into the respective education systems of the countries under study, offering special education needs services and support in mainstream education with the aim of including as many children into mainstream education as possible. Even though the education systems are comparable, the approaches between the countries under study are slightly different. Denmark and Sweden have passed several policies specifically geared towards special education needs, while Finland incorporates this more in general education policy.

**Conclusion:**

All countries under study have incorporated the values of the Universal Declaration of Human Rights and the Convention on the Rights of Persons with Disabilities in their respective education systems while emphasising the need to include as many children in the mainstream system as possible.

## Introduction

Autism Spectrum Conditions (ASCs, henceforth referred to as autism) are a set of neurodevelopmental conditions characterised by difficulties in communication, social interaction, and unusually narrow interests and/or repetitive behavioural patterns, starting in early childhood and continuing throughout life [1, 2]. The global prevalence of autism is estimated to be 1 percent of the population based on a review of prevalence studies across the world [3] with a male-to-female ratio between 3:1 and 4:1 [3, 4]. Autism is associated with adverse educational and employment outcomes, and many have significant health needs [3, 5, 6].

Early identification of autism and subsequent personalised help if needed in early life have been shown to benefit autistic people by improving language development, as well as behavioural and cognitive skills [7]. A key part of these therapies lies with the approach taken towards the child’s educational and developmental needs. The evidence base for special educational needs (SEN) support has been reported previously [8]. It was identified that support in the areas of ﻿cognition and learning, social, emotional and mental health, and communication and interaction can lead to significant benefits for the development of children with SEN, while also acknowledging that the SEN for autistic children may differ significantly per person.

Grindal and colleagues [9] describe four overarching educational approaches that schools can adopt: a) exclusion, which indicates an environment where children are denied access to education in any way; b) segregation, which happens when children with SEN are being educated in a separated environment; c) integration, which describes the environment where children with SEN are included in mainstream education, yet have to completely adapt to its standardised requirements; and d) inclusion (also known as inclusive education), which entails going beyond simply integrating children with SEN into mainstream education, to a process of systemic educational reform with a vision of providing equitable learning experiences for all children. They find that inclusive education conveys “clear and consistent evidence [of] substantial short- and long-term benefits for children with and without disabilities” [9]. When looking at children with disabilities specifically, they reported improved social and cognitive development, along with better integration into post-secondary education or employment.

The evidence base for the importance of equal and inclusive education for autistic children has steadily been reflected in the production of global, and more specifically European-wide, educational policies supporting the rights of autistic children in education. The crucial policy that protects and promotes the rights of autistic children was produced by the United Nations in the Universal Declaration of Human Rights (UDHR) [10], which states that everyone has a fundamental human right to an education “directed to the full development of the human personality”. After its ratification, it became the foundation of human rights policy, strategies, and actions in decades to come. It was followed up by the Convention on the Rights of Persons with Disabilities (CRPD) [11] which declares that “persons with disabilities can access an inclusive, quality, and free primary and secondary education on an equal basis with others in the communities in which they live”.

Even though the rights of people with disabilities had already been declared in the UDHR, the CRPD produced clear guidelines and rules that adopters had to adhere to. Consequently, it had a significant impact on both international strategies and national policies that sought to address the conditions for people with disabilities. Whilst the CRPD has been signed by all Member States of the European Union (EU), the competence needed to implement the values covering the educational rights of autistic people lies exclusively with the individual EU Member States. This is the result of the ratification of the Treaty on the Functioning of the European Union [12], which delegated the competence to regulate the education system (thus also the inclusion of children with SEN in that system) completely to the EU Member States.

Here, we will map the autism and SEN policies aimed at children under the age of 18 in the Nordic countries, namely: Denmark (5.7 million people), Sweden (10.1 million people), and Finland (5.5 million people) [13]. We aim to investigate how these Nordic EU countries approached the implementation of an education policy that promotes the rights of an autistic child to a fair and inclusive education. We will examine this by mapping SEN and disability policies in the context of key policy documents such as the UDHR and CRPD. Furthermore, this paper aims to specifically investigate how and to what extent the concept of inclusive education is implemented in national legislation. Inclusive education can have significant benefits for autistic children [8, 9] and the need for inclusive education to be introduced and developed in the national education systems has been endorsed at the international level as well in the Salamanca Statement [14] and more specifically in the CRPD [11]. Finally, an overview of the educational structure of the three countries under study is included in Additional file 1.

This work is part of a larger project of the European Consortium for Autism Researchers in Education (EDUCAUS) with an overarching aim of a systematic comparison of policy across all EU countries against the vision of an education system which supports autistic children to fulfil their potential. Like the previous work by EDUCAUS [15] (van Kessel R, Roman-Urrestarazu A, Ruigrok A, Holt R, Commers M, Hoekstra RA, et al. Autism and Family Involvement in the Right to Education in the EU:Policy Mapping and Scoping Review of Nordic Countries Denmark, Finland, and Sweden. Forthcoming), this was done by investigating how the values set out in UN documents like the UDHR and CRPD were translated into national education policy over time. We chose to focus on the abovementioned countries because of their shared geographical and cultural characteristics, as well as similar policy values, which should make for an equal comparison between the countries. Furthermore, these three countries account for 4.2% of the total EU population (512.6 million people) [13] influenced by autism and SEN policies.

## Methods

Previous work by Roleska and Roman-Urrestarazu and van Kessel and Roman-Urrestarazu established the theoretical framework and validated the methodology that we used in this policy mapping exercise [15] (van Kessel R, Roman-Urrestarazu A, Ruigrok A, Holt R, Commers M, Hoekstra RA, et al. Autism and Family Involvement in the Right to Education in the EU:Policy Mapping and Scoping Review of Nordic Countries Denmark, Finland, and Sweden. Forthcoming). The scoping review methodology allows for swift mapping of the key concepts underpinning a wide research area. This methodology is especially suitable for investigating complex matters that have not been comprehensively reviewed [16, 17]. Additionally, this scoping review and mapping project was performed through the means of a policy path dependence analysis [18]. This methodology is particularly useful for investigating the development of policy based on preceding legislation (such as the UDHR and CRPD) combined with conditional factors [18]. It also amalgamates competing ideas and values, which allows for the examination of interactions among different countries as well as how they follow supranational guidance (e.g. United Nations or EU guidance).

Because there is no single, representative data source in the EU with regards to autism and SEN policy, we adopted a qualitative modular approach to legislative and policy work across the different educational policy layers of analysis (Danish, Swedish, and Finnish specific). This approach divided the searches into two categories: (1) legislation and policy, and (2) scientific literature. Both categories were independently searched in duplication by two of the main authors. By independently executing the search strategy and comparing results afterwards, replicability could be warranted, thus increasing the reliability of the work [19]. After the searches were completed, the results were compared and synthesised into a single dataset, from which further analysis was derived. We used the PRISMA framework to report our findings [20].

### Theoretical framework for data analysis and path dependency

An analysis of policy path interdependency was carried out drawing on past and current international, EU, and national policies in the field of SEN and autism from 1948 up to date as part of the EDUCAUS project. Path dependence approaches allow the identification of policymaking patterns and establish influences and interrelations among policies in linear layers of temporality [21]. It also enables policy process-tracing, which (1) aims to explain what factors are present in critical policy junctures, (2) aims to create a reference framework and depict how decision processes come to conclusions, and (3) aims to describe how behaviour that takes place in different stakeholders as a response to external factors (e.g. a change in the policy environment) affects different institutional arrangements [22, 23]. In this case, the UDHR is taken as the starting point, a milestone document that influenced the creation and the content of EU and national policies. We used a timeline to show connection and overlap between policies to enable further analysis. This enabled the interpretation of policy creation as historical sequences and patterns and allowed for the identification of path dependence [21]. Current disability, inclusion, and autism policies are a result of previous events that were tracked with the use of this framework. All policies were analysed by identifying their input in the field of education, advantages and disadvantages, and their relation to other policies.

### Eligibility criteria

In order to remain consistent with the work previously done by EDUCAUS (van Kessel R, Roman-Urrestarazu A, Ruigrok A, Holt R, Commers M, Hoekstra RA, et al. Autism and Family Involvement in the Right to Education in the EU:Policy Mapping and Scoping Review of Nordic Countries Denmark, Finland, and Sweden. Forthcoming), the eligibility criteria displayed in Table 1 were used during the data collection of this study.

**Table 1:** A summary of the eligibility criteria

Inclusion criteria	Exclusion criteria
-Scope related to the right to education, the national education systems, disability laws, inclusion, and special education needs;	-Policies by non-governmental organisations.
-Aimed at those under 18 years of age;	
-Documents drafted by a governmental institution;	
-Publication date after 1948.	

### Data collection and search strategy

The first step in this policy mapping was to review and extract relevant policies and legislation that address the right to education of autistic people directly from original governmental sources. Several databases were used in the collection of data. The national Danish policy website (https://www.retsinformation.dk), Swedish policy repositories (https://beta.lagrummet.se/ and https://www.government.se/), and the Finnish policy repository (FinLex; https://www.finlex.fi/en/) were used for the retrieval of Danish, Swedish, and Finnish policy documents respectively. Additionally, the EU database for national policy (N-Lex; http://eur-lex.europa.eu/n-lex/) was also used to search for the national governmental documents. No limit was put on language and no time limit was used during the searches, as the goal was to create a timeline of policy development, implementation, and interaction. As a result, these search criteria allowed our search strategy to find relevant constitutions that date prior to 1948 as well. In order to adequately gauge the impact that the UDHR and subsequent policy have had on national policy, it was important to include these national constitutions as reference points.

The second step was to develop a multi-layered search strategy for electronic databases (PubMed and Google Scholar). A selection of key terms was created to use as the foundation of the search terms: “autism; disability; SEN; education; law; policy; right to education; special needs; special education; inclusive education”. Next, the academic databases PubMed and Google Scholar were searched using the following combinations of search terms: “autism & disability”; “autism & SEN”; “autism & education”; “autism & law”; “autism & policy”; “SEN & disability”; “SEN & law”; “SEN & policy”; “disability & law”; “disability & policy”. The final search query is shown in Table 2, along with its constituent terms. The national policy depositories were searched using the separate key terms, as combining the search terms yielded little results. The third step consisted of merging policy and academic publications according to the eligibility criteria.

**Table 2:** The build-up of the final search query for academic databases

	Search query
Term 1	((((((((((autism & law) OR autism & policy) OR autism & SEN) OR autism & education) OR autism & disability) OR SEN & policy) OR SEN & law) OR disability & law) OR disability & policy))
Term 2	((Denmark OR Sweden OR Finland))
Final Query	((((((((((autism & law) OR autism & policy) OR autism & SEN) OR autism & education) OR autism & disability) OR SEN & policy) OR SEN & law) OR disability & law) OR disability & policy)) AND ((Denmark OR Sweden OR Finland))

The fourth step was to acquire further information through searching reference lists of key articles (e.g. scientific articles, policy documents, governmental documents) and grey literature. Policy documents and governmental strategies in the countries under study were compared to the already mapped EU disability and educational policy. In case documents were not present, general disability policies and legislation were analysed. The data collection was built on the appraisal of three searches: one for every country under study. The final step was to merge the three searches into one single data repository for the purpose of this scoping review and to compare it to the already mapped policy of the United Nations and the EU for further analysis.

### Data analysis

#### Inter-rater reliability

Since the data collection of this paper is performed in duplication by the two main authors, it is crucial to determine inter-rater reliability. This was done by determining Cohen’s Kappa for each country under study separately. The analysis was performed using R [24], particularly using the ‘psych’ package [25].

#### Determination of path dependency

After the search strategy was completed, the gathered data was compared to the data that was already gathered on UN and EU policy in the previous work of EDUCAUS (van Kessel R, Roman-Urrestarazu A, Ruigrok A, Holt R, Commers M, Hoekstra RA, et al. Autism and Family Involvement in the Right to Education in the EU:Policy Mapping and Scoping Review of Nordic Countries Denmark, Finland, and Sweden. Forthcoming). As a result, the extent to which the values of international policies are integrated into the national policies could be established. An overview of international policies and their respective values is provided in Additional file 2.

## Results

**Fig. 1:**
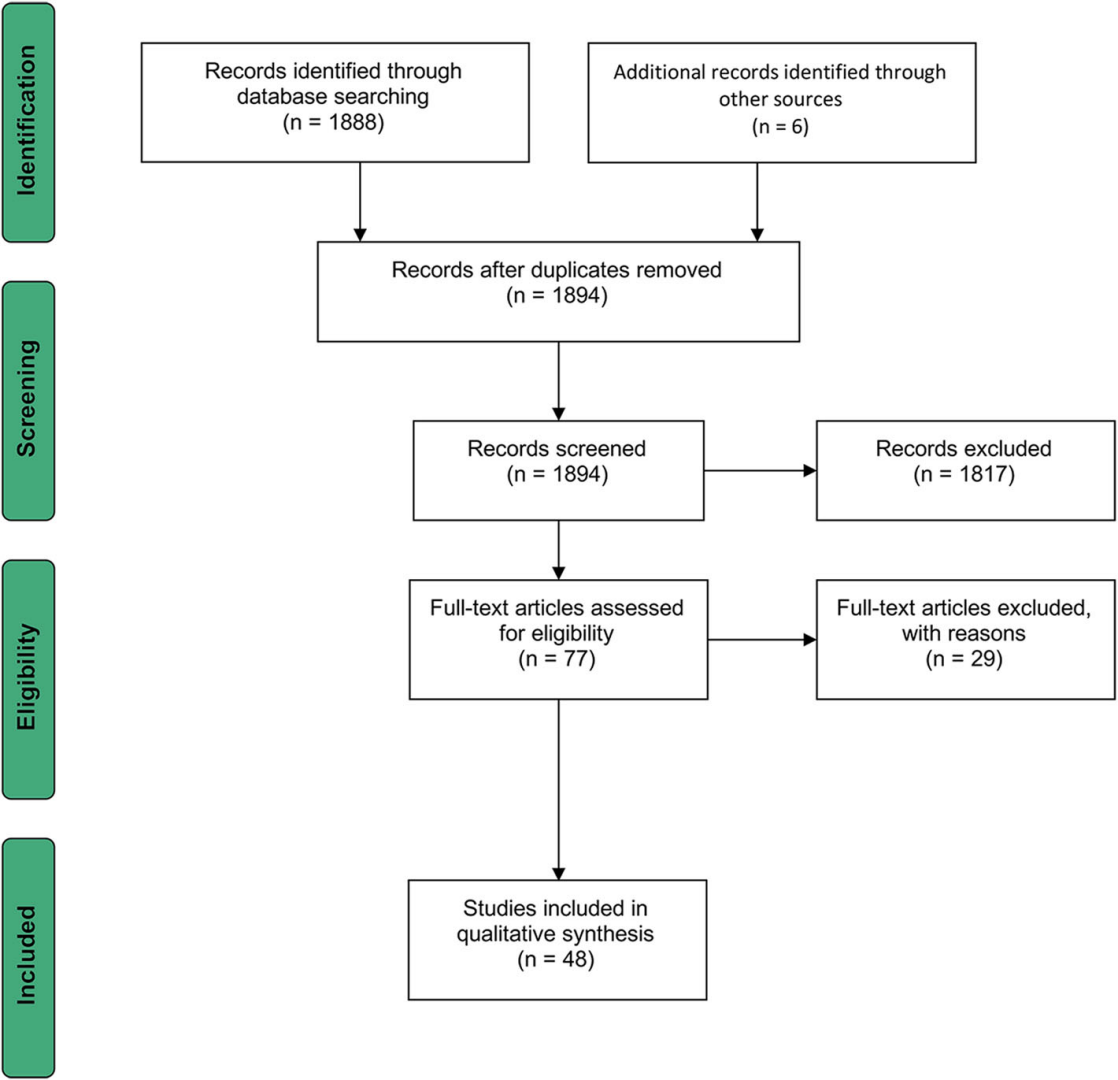
An overview of the data collection process using a PRISMA flowchart

**Fig. 2:**
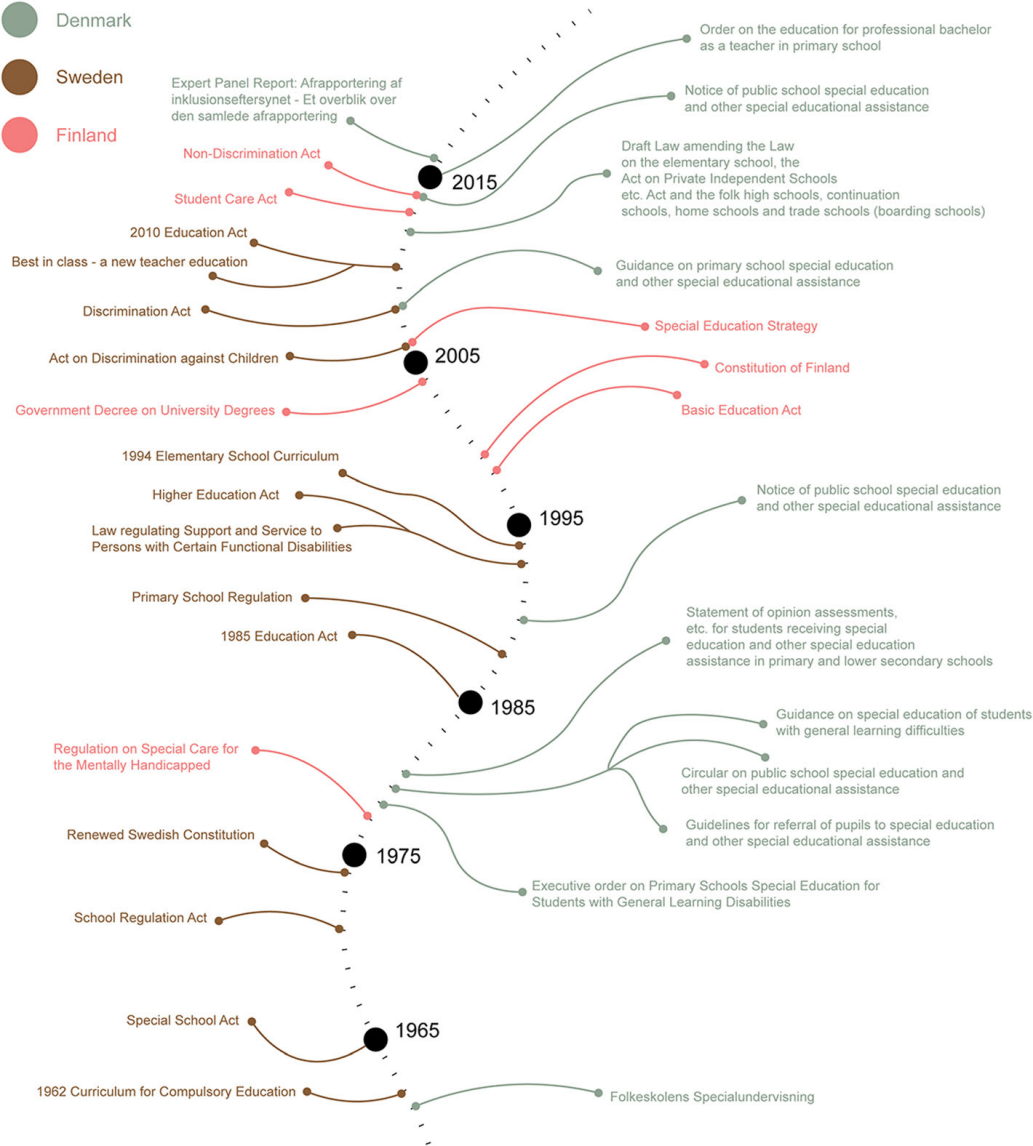
A chronological overview of Danish, Swedish, and Finnish policies with regard to SEN and autism

We identified 1888 sources (437 for Denmark, 1032 for Sweden, 419 for Finland) through database searching and 6 through other sources. No duplicates were identified; therefore, 1894 sources were analysed against the eligibility criteria. After reviewing abstracts, 77 sources were considered eligible for full-text screening. Examples of excluded items included a legislative piece on how education for adults with SEN is regulated, a regulation on addressing deaf-blind and visually impaired children specifically, and legislation that only applied to children with SEN outside the educational institution. Even though these items matched the search criteria, they fall outside the scope of this report. The full-text screening concluded in the further exclusion of 29 articles, with a difference in scope, lack of relevance, and unavailability of the full text being the most common factors. The remaining 48 articles (43 policy documents and 5 scientific sources) were included in this review. Also, the Danish policy repository only included policy post-1985. Therefore, we used a non-scientific database (Google) to search for documents that reported on Danish education policy pre-1985. In doing so, we identified 1 report by the Danish Ministry of Education that is considered grey literature, which is represented in the 6 sources mentioned above. A PRISMA flowchart illustrates the entire process in Figs. 1 and 2. Additionally, since the search was independently conducted by the two main authors, Cohen’s Kappa was used to ensure inter-reliability [26] in the database searches. This was done by appraising the searches and outcomes of each country under study separately (Additional file 3). The results of this analysis are shown in Table 3. In order to account for the lower outcomes for Sweden and Finland, country experts were involved to ensure the completeness and correctness of the policy analysis.

**Table 3:** An analysis of inter-reliability of the policy database searches using Cohen’s Kappa

		Denmark	Sweden	Finland
Investigator 1	Eligible	33	25	13
	Not eligible	404	1007	406
Investigator 2	Eligible	38	27	20
	Not eligible	399	1005	399
Total included		25	12	6
Total excluded		412	1020	413
Cohen’s Kappa		0.81	0.51	0.39

### Denmark

Denmark has adopted numerous policies that have shaped their education system to incorporate and include children with SEN. A full summary with descriptions of policies is included in Additional file 4.

The development of the Danish education system for SEN can be divided into two parts, based on the policies included. On the one hand, documents up until 1979 had a general theme of establishing a definition for SEN and clarifying that children with SEN should be identified as early as possible in order to be able to address their conditions as adequately as possible. More specifically, the constitution [27] specified access to education for children with SEN and ‘Folkenskolens Specialundervisning’ [28] recognised that children with SEN have particular needs that need to be addressed. Subsequently, the Executive Order on Primary Schools Special Education for Students with General Learning Disabilities [29] set out a number of options for the educational environment for these children ranging from having all teaching take place within a mainstream classroom but with additional support (i.e. an inclusive approach); to being educated partly within mainstream classrooms and partly within the special needs classroom (i.e. an integrative approach); and finally, to being solely educated separately from their peers, which could take place either within mainstream or special schools (i.e. a segregate approach). The accompanying circular on public school special education and other special educational assistance [30] further developed these options by prescribing class sizes for SEN education and enabling the extension of mandatory education when necessary.

On the other hand, documents from 1980 onward generally had the theme of shaping the education system to better include the children with SEN, so that they are not isolated in special education, away from their typical peers. The first step towards this was making sure that the progress of children with SEN was able to be measured without being compared to their typical peers. In the Statement of opinion assessments, etc. for students receiving special education and other special education assistance in primary and lower secondary schools, it is explained that, for children with SEN, a separate declaration can be made available that is indicative of them achieving their progress in a certain topic [31]. Next, all existing legislation on SEN education was unified into a single document: The Special Education and Other Special Assistance in Folkeskolen [32]. It ‘re-humanised’ the children with SEN, as it specifically emphasised that the needs of a child should be focused on, rather than the diagnosis, and that children with SEN should no longer be viewed in categories. It also stressed the role of the pedagogical-psychological service (PPR) in deciding if a student needs special education or other special assistance. The two last policies in this theme both aimed at fostering a more inclusive environment, moving away from segregation as much as possible. The measures set out in the Guidance on primary school special education and other special educational assistance [33] encapsulate the essence of truly inclusive education of children with SEN. It is not just simply legislating for the presence of children with SEN into mainstream education, but also a change across the whole culture and organisation of the school that embraces the whole worth of the child for the benefit of that child, their peers, and the school. Finally, the Folkeskole Law amending the Law on the elementary school, the Act on Private Independent Schools etc. Act and the folk high schools, continuation schools, home schools and trade schools (boarding schools) recognised the increase of children in special education [34]. Its aim was to reduce the number of children admitted to special education and the provisions set out to achieve that were (1) redefining when a child would be considered a child with SEN; and (2) giving the municipality and school’s director a more decisive role in judging when a child is to be placed in special education.

One notable detail about access to free education is that it has been a legal right of all children in Denmark as far back as 1814 and compulsory since 1855 [35]. In 1814, education access covered only 7 years of education (from age 6–7 years to 14 years); however, the coverage has been extended through the course of the 20th Century to apply to children from age 5–6 years to the age of 16 years [36]. This right was later re-affirmed in the Danish Constitution of 1953 [27].

It is mentioned above that the Special Education and Other Special Assistance in Folkeskolen unified previous legislation on SEN education. These prior documents were the first available legal documents to recognise the specific requirements of children with specific conditions. It started with a report on special education from a commission under the Danish Ministry of Education concerning students with intellectual disabilities and students with reading, speech, visual, or hearing problems [28]. This report was followed up with specific circulars [37–46] concerning the education of students not able to attend the regular school due to behavioural and psychological conditions (1972), dyslexia (1974), deaf and hearing-impaired, blindness and disability involving vision, physical handicap, language, and speech problems (all in 1979).

Grants for SEN are available in Denmark for additional teaching hours and materials to support the pupils, practical assistance to aid assimilation into school life more broadly, and for counselling support for parents and teachers [47]. Referral to regional authorities is generally done by the school, although this process can be triggered by the parents or healthcare staff. Where necessary, assistance in determining the individual needs of the child are available from a national knowledge and advisory service. Provision is also laid out for children whose needs would be best served in a school outside of their locality, with a funding arrangement between the municipality where they are resident and that of the school.

Given the responsibilities of the primary school teachers to address SEN in class, the education for teachers was examined as well. The Order on the education for professional bachelor as a teacher in primary school gives a complete overview on the competences that a primary school teacher should be proficient in [48]. From these competencies, three focus on or involve the education of children with SEN, which are respectively called ‘student learning and development’, ‘general teaching competence’, and ‘special education’. Whereas the first two tracks involve the general ability of a teacher to recognise and act upon the behaviour, capabilities, and environment of a child, the special education track sets out specific details for teachers to learn in order to best address SEN in their classroom environment (e.g. reasoned planning and executing an adapted teaching strategy).

Despite progress, and although the Danish Parliament argued for the 2012 ‘inclusion-law’ [34] in light of the Salamanca Statement [14], the ‘inclusion law’ was also motivated by economic considerations. While the changes in SEN policy in Denmark over the years have been for the most part motivated by new insight into the needs of SEN students, this weakened in the wake of the 2008 financial crisis and the coinciding public sector reform in Denmark that shifted all of the economic costs of SEN services solely to the local municipality. The Executive Orders and Guidance documents in 2012–2014 accompanying the Folkeskole law [49–51] that dropped the crucial provision that a decision on a student’s need for special education service and assistance should never be based on economic resources, placed the over-all decision-making power with the school principal (on behalf of the municipal authorities) and reduced the role of the PPR, parents, and teacher in the decision process must also be viewed in an economic light apart from educational and didactic considerations.

The 2012 ‘inclusion law’ sparked a strong, and ongoing, public debate on its effects, in response to which the government appointed an expert panel to characterise the student population affected by the transition to inclusion, identify key problems and make recommendations for practical implementation of inclusion. In the expert report published in 2016 [52], the panel’s overall impression was that inclusion had inaugurated positive trends in students’ overall welfare but substantial challenges remained to be met. The report further detailed 8 major challenge areas and many associated recommendations around strengthening inclusive learning environments; focusing on students’ needs in learning and well-being and engaging students; better prioritization of efforts and resources at all levels; strengthening proactive rather than reactive measures; improving access to professional knowledge and help such as PPR; strengthening competencies of teaching staff in working with children with special needs; and strengthening parental involvement and responsibility.

Although these recent events illustrate the potential for SEN policy setbacks in real time, in the larger picture, Denmark has made significant steps in moving from a segregated approach to educating children with SEN towards a more inclusive approach. Free education for all children has always been implemented in the Danish system; a clear definition of SEN was established early on, as well as the right that these children should receive an education like other children; and school systems and teacher education were changed incrementally over time to better support children with SEN.

### Sweden

Sweden has adopted several elaborate policies in order to form its education system to its current state. An overview of all included policies with their descriptions has been added in Additional file 5.

Compulsory education in Sweden was already implemented before the implementation of the UDHR in 1948. The universal Elementary school was introduced as far back as 1842 and 6 years of schooling was made compulsory for all children in 1882 [53]. This implied that pupils from all social strata entered the school, including children with special education needs and/or developmental disabilities, although they were offered only minimum courses (i.e. a very short period of schooling). The first step in developing the Swedish education system after the ratification of the UDHR by the UN was to clearly establish the rights of children with SEN. This was done firstly by the 1962 Curriculum for Compulsory Education [54, 55], which tasked schools to actively contribute to the development of their children into independent, individual, and harmonious adults as much as possible. Additionally, it stressed the need for children with SEN to attend special classes and established eight of these for children with disabilities to attend. The Special School Act [56] subsequently specified developmental delay as a condition that required SEN services to be distributed to. The Swedish Constitution [57] solidified the right to free and accessible education for all children covered by compulsory education and put the institutions in charge of taking care of the children during their stay.

The first mention of an integrative or inclusive school system came right before the implementation of the Swedish Constitution in the School Regulation Act [58], which also addressed the roles of the teachers and specialised equipment in attending to the SEN of children. This was later elaborated by the 1985 Education Act [59], which also laid down the foundation for the current education system. The Act itself covers a lot of different aspects of the education system, most notably (1) right and access to education; (2) the option of special education only for children unable to attend mainstream education due to the severity of their condition; (3) provisions specifically for developmentally delayed children; and (4) the notion that children with autism specifically are included in the scope of legislation that focuses on children with intellectual/learning conditions. Additionally, the Education Act regulated funding for primary, secondary, and special education. It states that the municipalities are the principles of the public schools in their respective areas. This also implies that the financing of these institutions is regulated through governmental pathways. With regard to the provision of SEN support, it also explains that there is funding available for municipalities to use, as well as that the municipalities have the right to be reimbursed by the state for their expenditures on SEN support. The Primary School Regulation [60], which closely followed the 1985 Education Act, further elaborated on SEN, SEN services, and special education. It laid out that additional support needs to be provided for children that have difficulties at school and should ideally be given in the regular environment that the child would belong in, though it also acknowledged several groups that special classes can be arranged for.

Afterwards, in the 1994 Elementary School Curriculum, the necessity to meet the rights and needs of children in school is stressed again. What this document expands on, though, is the role of the teachers, specifying that they should consider the needs, requirements, experiences, and thoughts of a child while teaching them. Finally, a new Education Act was implemented in 2010 [61]. Compared to the previous Act (which it still built upon), the way autism was handled changed slightly, i.e. it was no longer being treated identically to intellectual disabilities in all cases, but only when an intellectual disability is actually present. According to the Act, everyone should be included in mainstream schooling and placement in special teaching groups should be used only as a provision of last resort. Moreover, the Act prescribes that the student health should become a central activity with a strengthened mandate to prevent the emergence of problems and promote student health and welfare.

The environment for children with autism and their families received some developments over the years as well, starting with the Law regulating Support and Service to Persons with Certain Functional Disabilities [62]. Its contents are focused on improving the environment in which the children with autism live, which, by extension, can have positive effects on their educational performance. It creates opportunities for parents/guardians to relax by providing services that temporarily take care of their child with autism. Alternatively, it also puts measures in place that enables a child with autism to be placed elsewhere if it cannot live at home due to its condition. The Act on Discrimination against Children and the Discrimination Act [63, 64] predominantly addressed stigma and discrimination on the basis of disability. Their scope includes education, making it so that children with disabilities have as equal an opportunity as possible when attending mainstream education. The only difference between the two Acts is that the Discrimination Act expanded the scope of the Act on Discrimination against Children.

Since teachers are predominantly responsible for addressing the SEN of their children in the classroom, their education was investigated as well. The Higher Education Ordinance sets out the requirements for teacher training at all levels [65]. In terms of addressing SEN, the curriculum recognizes two different types of special educators: Special Education Needs Coordinators (SENCOs) and special education teachers. Göransson, Lindqvist, and Nilhom explain that the education of both types of special educators is the same in many aspects [66]. According to them, both education paths should lead to a skillset that is needed to work for and with children with SEN, as well as knowledge to develop and lead educational work with the goal to address the needs of all children. They further elaborate that the difference between the two types is that special education teachers are schooled to work in compulsory schools while SENCOs are schooled to work in preschools as well as compulsory schools with the child’s whole education environment. In terms of teacher education, this is reflected by the fact that special education teachers receive more individual-centred learning goals (e.g. knowledge of assessment and grading as well as language and conceptual development), while the education for SENCOs focuses on schools’ organisation and learning environment. They finally note that both groups are trained to be able to work in schools for children with intellectual disability. Later on, when implementing the ‘Best in class—a new teacher education’ proposal [67], mainstream teacher training was changed. Whereas previously teacher training applied to all of mainstream education, it is now subdivided into four categories: pre-primary education, primary education, subject education, and vocational education, each specialising on that specific part of the educational trajectory.

Ultimately, SEN services have existed in the Swedish education system since before the adoption of the UDHR. They also recognised and classified autism as a condition on par with developmental disorders as early as 1985 and later differentiated it from intellectual disabilities in 2010. Additionally, the Swedish system is structured in its approach towards SEN. According to the mapped legislation, SEN should first be addressed in mainstream classrooms to the best of the teacher’ abilities. Only when it is no longer feasible to keep a child with SEN in a mainstream classroom, can a transferral to special education be considered. Also, since the municipalities are in charge of their respective primary and secondary schools, the financing of SEN support in these schools is state-governed.

### Finland

Finland has adopted various policies since the UDHR was implemented by the UN. A synopsis of the policies included in Additional file 6.

When the UDHR was implemented, Finland already had some resemblance of an education system in place [68]. The first elementary schools were established in 1866, though home and church were responsible for basic education (e.g. reading and writing) before a child would be accepted in an elementary school, which was completely free. Compulsory education was introduced shortly after the First World War, in 1921. After the Second World War, education of children started with elementary school for everybody. Upon completion, there was a choice between two educational trajectories: upper comprehensive schooling, which led to studying in university; or senior primary schooling, which led to studying in vocational schools.

The first notice of allowing children with SEN to follow education came in the Regulation on Special Care for the Mentally Handicapped [69], which allowed for children that were unable to attend mainstream education to receive training separately until the end of their compulsory school age. Though in order for this to apply, they would need to be assessed by trained professionals in order to gauge their possibilities and capabilities. A more inclusive approach to the education of children with SEN came with the ratification of the Basic Education Act [70]. This Act addressed many different aspects of education and the incorporation of children with SEN in the education system. It sets out goals for teaching, clarifies what SEN services are and that, when possible, they should be provided in a mainstream classroom, sets out conditions under which a child may be partially or completely moved to special education, and explains what the personal plans for the organisation of teaching should include, along with whom should be included while drafting. Subsequently, the renewed Finnish Constitution [71] re-emphasised several basic human rights for children with SEN, such as that all children should be treated equally and their right to free basic education.

Over time, special education continued to grow in size, to the point that the Special Education Strategy [72] was drafted in order to create a proposal for a long-term strategy that would improve pre-primary and basic special education. In this strategy, it was proposed that the primary form of support would need to shift to an earlier support and prevention-based approach. The aim of this approach was to ﻿reinforce learning and growth and to prevent the magnification and/or escalation of problems relating to learning, development, or social interaction. Additionally, the Strategy urged for the content of special education to improve in a way that decisions on whether a child needs special education would become binding, including resources required, group size of the class, the adaptation of the syllabus, and the capacities of the child. However, it also recognises that it would be necessary to change the process of coming to a decision on SEN slightly, in a way that it should include pedagogic experts as well.

The final document that addressed the provision of services to children with SEN among others is the Student Care Act [73], which can be divided into three themes: its aims, allocating responsibilities, and the student health plan. The aims all refer back to the rights of the children set out in the previously covered documents. In terms of responsibilities, the municipalities in which the educational institution is located are allocated responsibility for the children, the involvement of psychologists or social workers, and, albeit optionally, provide support in organising additional services for educational institutions. Lastly, it introduced a student health plan, which includes details such as the amount of healthcare the child requires, the initiatives to protect the child from bullying, harassment, and a cooperation of different stakeholders that contribute to the child’s well-being. Even though it does not specifically target potential SEN of children, it can be useful to keep track of the additional health needs that many of the conditions that cause the SEN to require. As such, it has the potential to indirectly benefit the education of children with SEN.

With teachers being predominantly responsible for addressing the SEN of their children, it is crucial to investigate their training as well. The Government Decree on University Degrees [74] sets out the courses in which teachers in Finland are trained. During teacher training, aspiring teachers follow courses on each potential step of the education system: early and pre-primary education, basic education, special education, etc. As such, every teacher has at least a basic understanding of what SEN are and how to approach them in a classroom.

In short, the SEN policy in Finland is incorporated in general education policy. There are few policies aimed towards SEN or disability specifically, yet these topics are broadly covered in the overarching policies that regulate education (e.g. the Basic Education Act). Nevertheless, some form of equity is achieved through the referenced articles from the Constitution and the way SEN services are set up in the Acts. This is because legislation dictates that every child should be given the resources and services they need in order to develop themselves to their fullest. It is not specified how SEN services are financed in educational institutions. However, since educational institutions are funded by the state, it is implied that the provision of SEN services falls under this funding as well. Overall, as the Finnish basic education system is based on the philosophy of inclusion and all children are supported individually so that they can successfully complete their basic education.

## Discussion

This study aimed to map autism, SEN, and education policies that cover the right and access to education for autistic children, as well as the assistance provided during their education in Denmark, Sweden, and Finland. Additionally, it investigated how inclusion was integrated into respective national education policy using a policy path dependence analysis. This data was compared to the reference framework of UN and EU policy that was already available prior to this study (van Kessel R, Roman-Urrestarazu A, Ruigrok A, Holt R, Commers M, Hoekstra RA, et al. Autism and Family Involvement in the Right to Education in the EU:Policy Mapping and Scoping Review of Nordic Countries Denmark, Finland, and Sweden. Forthcoming). As a result, we mapped all relevant SEN policies that affect the education of autistic children as well as their universal right to education in an attempt to create a comprehensive report once the EU mapping exercise is complete with the aim of unveiling both bad and good practices when it comes to SEN education.

As already established in previous work (van Kessel R, Roman-Urrestarazu A, Ruigrok A, Holt R, Commers M, Hoekstra RA, et al. Autism and Family Involvement in the Right to Education in the EU:Policy Mapping and Scoping Review of Nordic Countries Denmark, Finland, and Sweden. Forthcoming), the UDHR marked a critical juncture for policy, both internationally and in the EU. When comparing the legislation adopted in the different policy layers to the Danish, Swedish, and Finnish policy environment, this also holds true for these countries. Notably, in the case of Denmark and Sweden, all children were already legally entitled to education prior to the ratification of the UDHR. Also, the approach towards the education of people with disabilities in Denmark and Sweden involved the ratification of policy that specifically addressed this topic on top of the coverage in basic education policy, while Finland has integrated its approach fully in basic education policy. Even though the approaches may differ slightly, the results are comparable.

All three countries have made significant efforts to adapt their education system to offer a place for all children (regardless of physical, mental, or social state), as well as to offer SEN services and support in mainstream education. That is up until the point it stops being in the child’s best interest, according to a group of experts that will assess each case individually, to attend mainstream education. In doing so, the UDHR’s values on the right to education are comprehensively considered in the three Nordic countries. Additionally, the values of UN and EU policy are reflected back in the national policy of these countries. Even though the international documents are not referenced in national policy, the measures that are taken are comparable in that similar outcomes are achieved: an improved education system that is appropriate and accessible for children with and without disability.

One of the aims of this study was to investigate to what extent the education system of the countries under study incorporated inclusive education. While the term itself is never mentioned in any of the legislative documents, the ideology is being adopted in all three countries. All three education systems aim to include as many children in their mainstream education as possible, with additional services, support, or equipment offered where required. This is reinforced by the notion that all educational institutions receive funding from their respective municipality, and by extension from their respective state, to accommodate as many children in their educational institution as possible, taking the health and educational needs of the child into account. Only if the child is burdened by their condition to such an extent that it is no longer feasible to attend mainstream education, will they be transferred to a special educational arrangement (e.g. special school setting, special teaching group). As such, it is safe to say that, while significant steps have already been made towards an inclusive environment, there is still a certain amount of segregation present in the Nordic education systems. Nevertheless, this is not necessarily a fault. While it is important for children with SEN to be included as much as possible in mainstream education for mutual benefit [9], it is also crucial that the education of typical children does not suffer from inclusion practices. As such, exclusion practices should be removed as soon as possible, but measures of segregation may still have a place in the current education system.

It is interesting to note that all three countries under study approach inclusive education from the perspective of the child with SEN. Even though the benefits for typical children to be in contact with children with SEN is well documented [9], it is notable how, for example, guidelines on the guidance of neurotypical children are left out of inclusive education policy, while they do play a crucial factor in inclusive education. Additionally, all three countries emphasise the role of the teacher in addressing SEN in the classroom. Therefore, we investigated the teacher training curricula as well and found that all three countries under study include courses on SEN and how to address them in a classroom. Unfortunately, the exact contents of the respective courses could not be reviewed at the time of writing this paper. As such, we can assert that teachers in Denmark, Sweden, and Finland all have at least a basic understanding of SEN and how to address them in a classroom.

It has to be noted that there are some contrasts between the three countries. As previously mentioned, Finland incorporates its SEN strategy more in general education policy, while Denmark and Sweden also passed several policies specifically geared towards SEN and disability policy. As a result, the environment in Finland has become less regulated and there are fewer guidelines to help schools formulate their SEN strategies. While this is not an inherently negative situation, it poses the risk that schools fill in the inevitable gaps in legislation locally without national guidance, which may result in a heterogeneous environment for children with SEN to study and develop. Also, while Danish and Swedish education policies strongly imply that their focal point is children, Finnish policy explicitly states so. Consequently, potential room for (mis)interpretation is removed in the application of these policies: children are the focal point and all measures taken by schools or other educational institutions should support them and their development. Additionally, even though all countries under study aim to address SEN for children with disabilities, Finland and Denmark operate without a clear definition of ‘disability’. Their policies generally refer to disabilities as ‘conditions that inhibit education’ and structurally state that teachers should ‘take measures to address the corresponding needs’, without going into detail as to what kind of needs should be met in what way. Furthermore, while Denmark and Finland predominantly put the educational institutions in charge of the decision-making processes that pertain to the child with SEN, Sweden includes family members in the process as well. The inclusion of family in the decision-making process is a factor that has already been reviewed in the previous work of EDUCAUS (van Kessel R, Roman-Urrestarazu A, Ruigrok A, Holt R, Commers M, Hoekstra RA, et al. Autism and Family Involvement in the Right to Education in the EU:Policy Mapping and Scoping Review of Nordic Countries Denmark, Finland, and Sweden. Forthcoming). One particular detail that should be noted in the Swedish system when considering how autism is addressed in education, is its disconnection from intellectual disabilities in recent times unless the child with autism actually has an intellectual disability. As a result, children with autism no longer run the risk of being sent to a special school based on their condition and have better access to education. Moreover, Finland has taken additional steps to support children with SEN. Instead of starting the process while the child attends primary school, it implemented measures that target pre-primary education and special education. In doing so, it aimed to create an educational environment in which learning and developmental conditions could be better addressed. Finally, in recent years, Danish policy has reconsidered the scope of SEN in an attempt to facilitate more children in mainstream classrooms. Now, children that require less than 9 teaching hours of additional support are not considered to be children with SEN and are to be assisted primarily by the teacher in charge. According to the 2012 ‘inclusion law’ [34], the child must be given additional education, support or personal assistance if needed to solve practical issues attending school, aiding their fundamental right to maximum development. Nevertheless, these measures put more responsibilities on the classroom teachers. The Danish government’s expert panel report in 2016 highlighted the strengthening of teachers’ competencies in working with children with special needs and access to relevant professional support as one of the key challenge areas in the implementation of inclusion.

This scoping review has some limitations that should be accounted for. Firstly, the results of this study cannot be generalized across countries, only within the three countries explored. Secondly, it is challenging to determine how the approaches to SEN are put into practice. Therefore, the results of this paper remain strictly theoretical. Thirdly, during the execution of the search strategy, the N-Lex database was unavailable for Denmark and Finland. A large advantage of using the N-Lex is that it will automatically translate an index term from English to the respective language of the country under investigation. However, since this service was unavailable, we relied on manual translations of index terms. Whilst all synonyms of the translated index term were included, it does not rule out the possibility that some meaning was lost in translation and that that might have affected the outcomes of this paper. To account for this limitation, Danish, Swedish, and Finnish experts were asked to assist in searching and interpreting the legislation for their respective countries. Fourthly, the Danish policy repository only included limited entries pre-1985. As a result, we had to rely on synopses of legislations that predate 1985 or had to acknowledge that the policy path dependence for Denmark would be incomplete. As it stands, there was only one completely unavailable policy document, namely the 1975 law on primary and lower secondary schools. Fifthly, only governmental documents were included in this research. Consequently, potential actions by NGOs were disregarded by this paper, unless their work was adopted in national legislation (e.g. the Charter for Persons with Autism, drafted by Autism-Europe and adopted by the European Parliament). Finally, the scope of this study was limited to autistic children. In other words, autistic adults that are part of the education system were not covered by the contents of this paper, even though they may experience similar challenges in their educational endeavours.

Lastly, there are some opportunities for further work as well. For instance, it would be beneficial to investigate how inclusion policies are translated into practice in the countries under study. At the time of writing this report, no such investigation has been done, even though it could provide significant improvements either in policy recommendations and/or in the education environment. Also, teacher training should be investigated beyond these Nordic countries. With inclusion becoming more prevalent in the EU, it is crucial that teachers have the knowledge, skills, and competences to adequately address SEN in their classrooms.

## Conclusion

This study provided insight into the SEN policy environment of Denmark, Sweden, and Finland. The values of the UDHR and CRPD were integrated into all education systems under study through national legislation. Mainstream schools offer SEN services and support until participation in mainstream education ceases to be in the child’s best interest due to the severity of their SEN. Also, the provision of SEN services is done exclusively through schools. There are no other institutions involved in providing these services. Inclusive education, while not mentioned specifically in national legislation, is a guiding factor in the education systems of the countries under study.

## Supplementary information

**Additional file 1.** Key characteristics of the Nordic education systems. This table gives a brief overview of the core components of the Nordic education systems.

**Additional file 2.** An overview of the specific policies and their values of the United Nations and European Union. This overview provides a list of the core values of the policies that involved human rights, autism, and special education that were implemented by the United Nations and the European Union.

**Additional file 3.** The source code used to calculate the inter-rater reliability. This source code shows the number of articles deemed eligible and non-eligible by both authors, how many they agreed on, and includes all details on how Cohen’s Kappa was calculated for all three countries to determine inter-rater reliability.

**Additional file 4.** A chronological overview and description of the Danish policies on education and SEN. A point by point overview of the implications of each respective policy that was analysed for Denmark.

**Additional file 5.** A chronological overview and description of the Swedish policies on education and SEN. A point by point overview of the implications of each respective policy that was analysed for Sweden.

**Additional file 6.** A chronological overview and description of the Finnish policies on education and SEN. A point by point overview of the implications of each respective policy that was analysed for Finland.

## Data Availability

While all data are publicly available, a list of used documents along with their source has been included.

## Abbreviations

ASCs: Autism spectrum conditions
CRPD: Convention on the Rights of Persons with Disabilities
EDUCAUS: European Consortium for Autism Researchers in Education
EU: European Union
NGOs: Non-governmental organisations
PPR: Pedagogical-psychological service
SEN: Special education needs
SENCOs: Special Education Needs Coordinators
UDHR: Universal Declaration of Human Rights

## References

[1] American Psychiatric Association. Diagnostic and statistical manual of mental disorders. 5th ed. Washington, DC; 2013.

[2] World Health Organisation. International Statistical Classification of Diseases and Related Health Problems 10th Revision [Internet]. 2016 [cited 2018 Nov 15]. Available from: http://apps.who.int/classifications/icd10/browse/2016/en#/F84.5

[3] Lai MC, Lombardo MV, Baron-Cohen S (2014). Autism. Lancet..

[4] Loomes R, Hull L, WPL M (2017). What is the male-to-female ratio in autism spectrum disorder? a systematic review and meta-analysis. J Am Acad Child Adolesc Psychiatry.

[5] Ganz M (2007). The lifetime distribution of the incremental societal costs of autism. Arch Pediatr Adolesc Med [Internet]..

[6] Shattuck PT, Narendorf SC, Cooper B, Sterzing PR, Wagner M, Taylor JL. Postsecondary education and employment among youth with an autism spectrum disorder. Pediatrics. 2012; Available from: http://pediatrics.aappublications.org/content/early/2012/05/09/peds.2011-2864.abstract. 10.1542/peds.2011-2864 PMC3362908 22585766

[7] Warren Z, McPheeters ML, Sathe N, Foss-Feig JH, Glasser A, Veenstra-Vander Weele J (2011). A Systematic Review of Early Intensive Intervention for Autism Spectrum Disorders. Pediatrics.

[8] Carroll J, Bradley L, Crawford H, Hannant P, Johnson H, Thompson A (2017). SEN support: A rapid evidence assessment.

[9] Grindal T, Hehir T, Freeman B, Lamoreau R, Borquaye Y, Burke S (2016). A summary of the research evidence on inclusive education.

[10] United Nations (1948). Universal Declaration of Human Rights [Internet].

[11] United Nations (2006). Convention on the rights of persons with disabilities [Internet].

[12] European Commission (2009). Treaty on the functioning of the European Union [Internet].

[13] Eurostat. Population change—demographic balance and crude rates at national level [Internet]. 2018 [cited 2018 Apr 30]. Available from: http://appsso.eurostat.ec.europa.eu/nui/show.do?dataset=demo_gind&lang=en

[14] UNESCO. The Salamanca Statement and Framework for Action on Special Needs Education [Internet]. 1994 [cited 2018 May 19]. Available from: http://www.unesco.org/education/pdf/SALAMA_E.PDF

[15] Roleska M, Roman-Urrestarazu A, Griffiths S, Ruigrok AN V., Holt R, van Kessel R, et al. Autism and the right to education in the EU: Policy mapping and scoping review of the United Kingdom, France, Poland and Spain. Jan Y-K, editor. PLoS One. 2018 [cited 2019 Apr 26];13(8):e0202336. Available from: http://dx.plos.org/10.1371/journal.pone.0202336 10.1371/journal.pone.0202336 PMC6116926 30161146

[16] Arksey H, O’Malley L (2005). Scoping studies: towards a methodological framework. Int J Soc Res Methodol..

[17] Levac D, Colquhoun H, O’Brien KK (2010). Scoping studies: advancing the methodology. Implement Sci..

[18] Mahoney J (2000). Path dependence in historical sociology. Theory Soc..

[19] Leung L. Validity, reliability, and generalizability in qualitative research. J Fam Med Prim care. 2015 [cited 2018 Oct 1];4(3):324–7. Available from: http://www.ncbi.nlm.nih.gov/pubmed/26288766 10.4103/2249-4863.161306 PMC4535087 26288766

[20] Moher D, Liberati A, Tetzlaff J, Altman DG, PRISMA Group (2009). Preferred reporting items for systematic reviews and meta-analyses: the PRISMA statement. BMJ.

[21] Mahoney J (2000). Path dependence in historical sociology. Theory Soc.

[22] Pierson P (2000). Increasing Returns, Path Dependence, and the Study of Politics. Am Polit Sci Rev.

[23] Collier R, Collier D. Shaping the political arena critical junctures, the labor movement, and regime dynamics in. 2002 [cited 2018 May 15]; Available from: https://cloudfront.escholarship.org/dist/prd/content/qt8qr1z7gc/qt8qr1z7gc.pdf?t=ok97a8

[24] R Core Team. R: A language and environment for statistical computing. Vienna; 2018. Available from: https://www.r-project.org/

[25] Revelle W. psych: Procedures for psychological, psychometric, and personality research. Evanston; 2018. Available from: https://cran.r-project.org/package=psych

[26] Cohen J. A coefficient of agreement for nominal scales. Educ Psychol Meas. 1960 [cited 2019 Aug 2];20(1):37–46. Available from: http://journals.sagepub.com/doi/10.1177/001316446002000104

[27] Folketing. The Constitutional Act of Denmark. Folketinget; 1953. Available from: https://www.thedanishparliament.dk/Publications/~/media/PDF/publikationer/English/The_Constitutional_Act_Of_Denmark_2013.pdf.ashx

[28] Danish Ministry of Education. Folkeskolens Specialundervisning. 1961. Available from: https://www.foxylex.dk/media/betaenkninger/Folkeskolens_specialundervisning.pdf

[29] Danish Ministry of Education. Executive Order on Primary Schools Special Education for Students with General Learning Disabilities. Danish Ministry of Education; 1978. Available from: https://www.retsinformation.dk/Forms/R0710.aspx?id=73318

[30] Danish Ministry of Education. Circular on public school special education and other special educational assistance. 1979. Available from: https://www.retsinformation.dk/Forms/R0710.aspx?id=74569

[31] Danish Ministry of Education. Statement of opinion assessments, etc. for students receiving special education and other special education assistance in primary and lower secondary schools. Danish Ministry of Education; 1980. Available from: https://www.retsinformation.dk/Forms/R0710.aspx?id=72959#

[32] Danish Ministry of Education. Notice of public school special education and other special educational assistance. 1990 [cited 2018 Dec 12]. Available from: https://www.retsinformation.dk/Forms/R0710.aspx?id=73491

[33] Danish Ministry of Education. Guidance on primary school special education and other special education assistance. 2008. Available from: https://www.retsinformation.dk/Forms/R0710.aspx?id=114197

[34] Danish Parliament. Folkeskole Law amending the Law on the elementary school, the Act on Private Independent Schools etc. Act and the folk high schools, continuation schools, home schools and trade schools (boarding schools). 2012 [cited 2018 Dec 12]. Available from: https://www.ft.dk/samling/20111/lovforslag/L103/som_vedtaget.htm#dok

[35] Danish Ministry of Education. About the Folkeskole. Danish Ministry of Education; 2018. Available from: http://eng.uvm.dk/primary-and-lower-secondary-education/the-folkeskole/about-the-folkeskole

[36] Danish Ministry of Education. Law on Primary Education. Aarhus University; 1975. Available from: https://danmarkshistorien.dk/leksikon-og-kilder/vis/materiale/lov-om-folkeskolen-26-juni-1975/

[37] Danish Ministry of Education. Circular on public school special education of pupils with behavioral problems and mental disorders. 1972 [cited 2018 Dec 12]. Available from: https://www.retsinformation.dk/Forms/R0710.aspx?id=74072

[38] Danish Ministry of Education. Circular on public school special education of learning disabled students. 1974 [cited 2018 Dec 12]. Available from: https://www.retsinformation.dk/Forms/R0710.aspx?id=74130

[39] Danish Ministry of Education. Order on notification obligations for voice disorders, severe cases of dyslexia and reading weakness. 1978 [cited 2018 Dec 12]. Available from: https://www.retsinformation.dk/Forms/R0710.aspx?id=73142

[40] Danish Ministry of Education. Circular on special education for students in the 8th-10th grade who need special support in the subject Danish. 1978 [cited 2018 Dec 12]. Available from: https://www.retsinformation.dk/Forms/R0710.aspx?id=74637

[41] Danish Ministry of Education. Circular on amending the Law on the elementary school, the law on special education for adults and amending the Law on leisure education, etc. 1978 [cited 2018 Dec 12]. Available from: https://www.retsinformation.dk/Forms/R0710.aspx?id=74179

[42] Danish Ministry of Education. Notice of public school special education of students with general learning disabilities. 1978 [cited 2018 Dec 12]. Available from: https://www.retsinformation.dk/Forms/R0710.aspx?id=73318

[43] Danish Ministry of Education. Notice of public school special education and other special educational assistance to students with language or speech problems. 1979 [cited 2018 Dec 12]. Available from: https://www.retsinformation.dk/Forms/R0710.aspx?id=73344

[44] Danish Ministry of Education. Notice of public school special education and other special educational assistance for the hearing impaired pupils. 1979 [cited 2018 Dec 12]. Available from: https://www.retsinformation.dk/Forms/R0710.aspx?id=73348

[45] Danish Ministry of Education. Notice of public school special education and other special educational assistance to visually impaired students [Internet]. 1979 [cited 2018 Dec 12]. Available from: https://www.retsinformation.dk/Forms/R0710.aspx?id=73347

[46] Danish Ministry of Education. Notice of public school special education and other special educational assistance for disabled students [Internet]. 1979 [cited 2018 Dec 12]. Available from: https://www.retsinformation.dk/Forms/R0710.aspx?id=73345

[47] Danish Ministry of Education. Notice of public school special education and other special educational assistance [Internet]. 2014 [cited 2018 Dec 12]. Available from: https://www.retsinformation.dk/Forms/R0710.aspx?id=163941

[48] Danish Ministry of Education and Research. Order on the education for professional bachelor as a teacher in primary school [Internet]. 2015. Available from: https://www.retsinformation.dk/Forms/R0710.aspx?id=174218

[49] Danish Ministry of Education. Guidance on primary school special education and other special educational assistance [Internet]. 2013 [cited 2019 Aug 22]. Available from: https://www.retsinformation.dk/Forms/R0710.aspx?id=145638

[50] Danish Ministry of Education. Executive Order: Notice of public school special education and other special educational assistance [Internet]. 2012. Available from: https://www.retsinformation.dk/Forms/R0710.aspx?id=141578

[51] Danish Ministry of Education. Executive Order on Primary Schools Special Education and Other Special Educational Assistance [Internet]. 2014. Available from: https://www.retsinformation.dk/Forms/R0710.aspx?id=163980

[52] Danish Ministry of Children, Education and GE. Afrapportering af inklusionseftersynet: Et overblik over den samlede afrapportering [Internet]. 2016 [cited 2019 Aug 27]. Available from: https://www.altinget.dk/misc/160511 PIXI version inklusion040516WEB.pdf

[53] Nordström S. Hjälpskolan och särskolan i Sverige t.o.m. 1921 [The School for the Back- ward and Severely Mentally Retarded in Sweden up to 1921]. [Internet]. Vol. 119, Årsböcker i svensk undervisningshistoria. Stockholm: Almqvist & Wiksell; 1968 [cited 2018 Dec 5]. Available from: http://www.tam-arkiv.se/share/proxy/alfresco-noauth/tam/content/workspace/SpacesStore/b9ef3ccf-f549-444a-a3d7-2f9dea8055e0/download/ASU_119.pdf

[54] Fischer M, Karlsson M, Nilsson T. The sooner the better? Compulsory Schooling Reforms in Sweden * [Internet]. 2014 [cited 2018 Dec 11]. Available from: https://www.ed.lu.se/media/ed/seminar_papers/reform_ulf_150212.pdf

[55] Swedish Ministry of Education (1962). Curriculum for Compulsory Education.

[56] Swedish Ministry of Education. Special School Regulation [Internet]. Sweden; 1965. Available from: https://beta.lagrummet.se/rinfo/publ/sfs/1965:478/konsolidering/1994-07-01

[57] Sveriges Riksdag. The Constitution of Sweden [Internet]. 1974. Available from: http://www.equalrightstrust.org/ertdocumentbank/CONSTITUTION OF SWEDEN.pdf

[58] Swedish Ministry of Education. School Regulation Act [Internet]. Sweden; 1971. Available from: https://beta.lagrummet.se/rinfo/publ/sfs/1971:235/konsolidering/1990-07-01

[59] Swedish Ministry of Education. Education Act [Internet]. Sweden; 1985. Available from: http://rkrattsbaser.gov.se/sfst?bet=1985:1100

[60] Swedish Ministry of Education. Primary School Regulation [Internet]. 1988 [cited 2018 Oct 9]. Available from: https://beta.lagrummet.se/rinfo/publ/sfs/1988:655/konsolidering/1994-07-01

[61] Swedish Ministry of Education. Education Act [Internet]. 2010 [cited 2018 Dec 12]. Available from: https://beta.lagrummet.se/rinfo/publ/sfs/2010:800/konsolidering/2017-11-01

[62] Swedish Ministry of Social Affairs. Law regulating support and service to persons with certain functional disabilities [Internet]. 1993 [cited 2018 Dec 12]. Available from: https://beta.lagrummet.se/rinfo/publ/sfs/1993:387/konsolidering/2017-07-01

[63] Swedish Ministry of Education. Act on Discrimination against Children [Internet]. 2006. Available from: http://rkrattsbaser.gov.se/sfst?bet=2006:67

[64] Swedish Ministry of Culture. Discrimination Act [Internet]. 2008 [cited 2018 Dec 12]. Available from: https://beta.lagrummet.se/rinfo/publ/sfs/2008:567/konsolidering/2017-07-01

[65] Swedish Ministry of Education. Higher Education Ordinance [Internet]. 1993. Available from: https://beta.lagrummet.se/rinfo/publ/sfs/1993:100/konsolidering/2017-10-01

[66] Göransson K, Lindqvist G, Nilholm C (2015). Voices of special educators in Sweden: A total-population study. Educ Res.

[67] Swedish Ministry of Education. Best in class—a new teacher education (Government Bill 2009/10:89) [Internet]. 2010. Available from: https://riksdagen.se/sv/dokument-lagar/dokument/proposition/bast-i-klassen%2D%2D-en-ny-lararutbildning_GX0389/html

[68] Kärnä E (1995). History of special education in Finland.pdf. Int J Spec Educ..

[69] Finnish Ministry of Social Affairs and Health. Regulation on Special Care for the Mentally Handicapped [Internet]. Oikeusministeriö, Edita Publishing Oy; 1977. Available from: https://www.finlex.fi/fi/laki/smur/1977/19770988?search%5Bkohdista%5D=koko&search%5Ball%5D=&search%5Bany%5D=koulutus opetus&search%5Bphrase%5D=&search%5Bwithout%5D=&search%5Btype%5D=tekstihaku

[70] Finnish Ministry of Education. Basic Education Act [Internet]. 1998. Available from: https://www.finlex.fi/en/laki/kaannokset/1998/en19980628.pdf

[71] Finnish Ministry of Justice. The Constitution of Finland [Internet]. 1999. Available from: https://www.finlex.fi/fi/laki/kaannokset/1999/en19990731.pdf

[72] Finnish Ministry of Education. Special Education Strategy [Internet]. 2006 [cited 2018 Nov 20]. Available from: http://julkaisut.valtioneuvosto.fi/bitstream/handle/10024/79498/tr47.pdf

[73] Finnish Ministry of Education, Finnish Ministry of Culture. Student Care Act [Internet]. 2013. Available from: https://www.finlex.fi/fi/laki/ajantasa/2013/20131287

[74] Finnish Ministry of Education. Government Decree on University Degrees (794/2004) [Internet]. 2004. Available from: https://www.finlex.fi/en/laki/kaannokset/2004/en20040794.pdf

